# Transcranial direct current stimulation (tDCS) in depression induces structural plasticity

**DOI:** 10.1038/s41598-023-29792-6

**Published:** 2023-02-17

**Authors:** Mayank A Jog, Cole Anderson, Antoni Kubicki, Michael Boucher, Amber Leaver, Gerhard Hellemann, Marco Iacoboni, Roger Woods, Katherine Narr

**Affiliations:** 1 Department of Neurology, University of California Los Angeles (UCLA), Los Angeles, CA 90095 USA; 2 Diagnostic Imaging Sciences Center, University of Washington, Seattle, WA 98195 USA; 3 Semel Institute for Neuroscience and Human Behavior, UCLA, Los Angeles, CA 90095 USA; 4 Department of Radiology, Northwestern University, Evanston, IL 60208 USA; 5 Department of Biostatistics, University of Alabama at Birmingham, Birmingham, AL 35294 USA; 6 Department of Psychiatry and Biobehavioral Sciences, UCLA, Los Angeles, CA 90095 USA

**Keywords:** Neuroscience, Psychiatric disorders

## Abstract

Transcranial direct current stimulation (tDCS) is a non-invasive neuromodulation technique involving administration of well-tolerated electrical current to the brain through scalp electrodes. TDCS may improve symptoms in neuropsychiatric disorders, but mixed results from recent clinical trials underscore the need to demonstrate that tDCS can modulate clinically relevant brain systems over time in patients. Here, we analyzed longitudinal structural MRI data from a randomized, double-blind, parallel-design clinical trial in depression (NCT03556124, N = 59) to investigate whether serial tDCS individually targeted to the left dorso-lateral prefrontal cortex (DLPFC) can induce neurostructural changes. Significant (FWEc *p* < 0.05) treatment-related gray matter changes were observed with active high-definition (HD) tDCS relative to sham tDCS within the left DLPFC stimulation target. No changes were observed with active conventional tDCS. A follow-up analysis within individual treatment groups revealed significant gray matter increases with active HD-tDCS in brain regions functionally connected with the stimulation target, including the bilateral DLPFC, bilateral posterior cingulate cortex, subgenual anterior cingulate cortex, and the right hippocampus, thalamus and left caudate brain regions. Integrity of blinding was verified, no significant differences in stimulation-related discomfort were observed between treatment groups, and tDCS treatments were not augmented by any other adjunct treatments. Overall, these results demonstrate that serial HD-tDCS leads to neurostructural changes at a predetermined brain target in depression and suggest that such plasticity effects may propagate over brain networks.

## Introduction

Transcranial direct current stimulation (tDCS) is an emerging neuromodulation technique that provides a safe, low-cost and non-invasive means of modulating the brain^1,2^. A typical tDCS setup uses a 9 V battery and non-invasive scalp electrodes to deliver mild, milliampere electric currents at a targeted brain region^3^. In cell culture as well as in *in-vivo* motor cortex experiments, tDCS electric currents have been shown to induce electric fields that, depending on the polarity of the applied stimulation, increase or decrease local neuronal activity^4,5^. These findings have provided the basis for research into clinical applications of tDCS, hypothesizing that tDCS-induced mitigation of pathologic neural activity will result in clinical improvement. Accordingly, tDCS treatment protocols targeting dysfunctional neural circuits in clinical disorders have been investigated for potential translation into clinical practice^2,6^.

Clinical trials investigating therapeutic effects of tDCS have shown mixed results. In major depression, Brunoni et al.^7^ observed significant mood improvement with tDCS treatment compared to placebo. However, another recent clinical trial did not demonstrate clinical superiority of active tDCS relative to sham in depression^8^. Similar mixed findings are also reported in schizophrenia studies investigating treatment of auditory verbal hallucinations with tDCS (^9,10^, see also review^6^). While disease heterogeneity and other systematic differences between study samples could contribute to mixed results, another factor could be the parameters of the tDCS treatment protocols themselves. For instance, a recent study employing intracerebral measurement electrodes in cadavers showed that only about 25% of the applied tDCS current reaches the cortex^11^ and recommended a substantial increase in the 2 mA electric currents typically employed in tDCS protocols to consistently elicit changes in neuronal activity. However, recent brain imaging studies have shown that significant neurophysiological changes can be induced by the 2 mA electric currents typically used in existing tDCS protocols^12,13^. These mixed results from methodological studies as well as clinical trials underscore the fundamental need to determine whether existing tDCS protocols can induce robust neurobiological changes *in-vivo*.

In this study, we investigated whether serial tDCS therapy can induce persistent changes in brain structure in depressed participants. Regional reductions of gray matter measured with MRI are a well-replicated feature of disease processes in depression^14,15^. At least one large-scale randomized clinical trial has reported that tDCS treatment is of a similar efficacy as standard pharmacotherapy^7^, and thus it is plausible that tDCS administration may affect or normalize altered brain structure in depressed participants. Additionally, in vitro and animal studies have shown that externally applied electric currents (such as in tDCS) can induce morphological changes in neural tissue^5,16^. Consequently, we hypothesized that active tDCS would induce significant changes in brain structure in depressed participants, relative to sham tDCS. Note that most tDCS studies in depression (including this trial) have utilized tDCS as the sole experimental therapeutic intervention^7,8^. The absence of any concomitant interventions, together with the inclusion of a placebo control group in our study design, enables a clearer determination of whether tDCS treatment alone can induce structural neuroplasticity. While tDCS in conjunction with physical or other active therapies has been reported to induce structural changes in other neurological populations^17,18^, the question of whether tDCS treatment itself can induce macroscopic changes in brain architecture has not yet been addressed.

**Figure 1:**
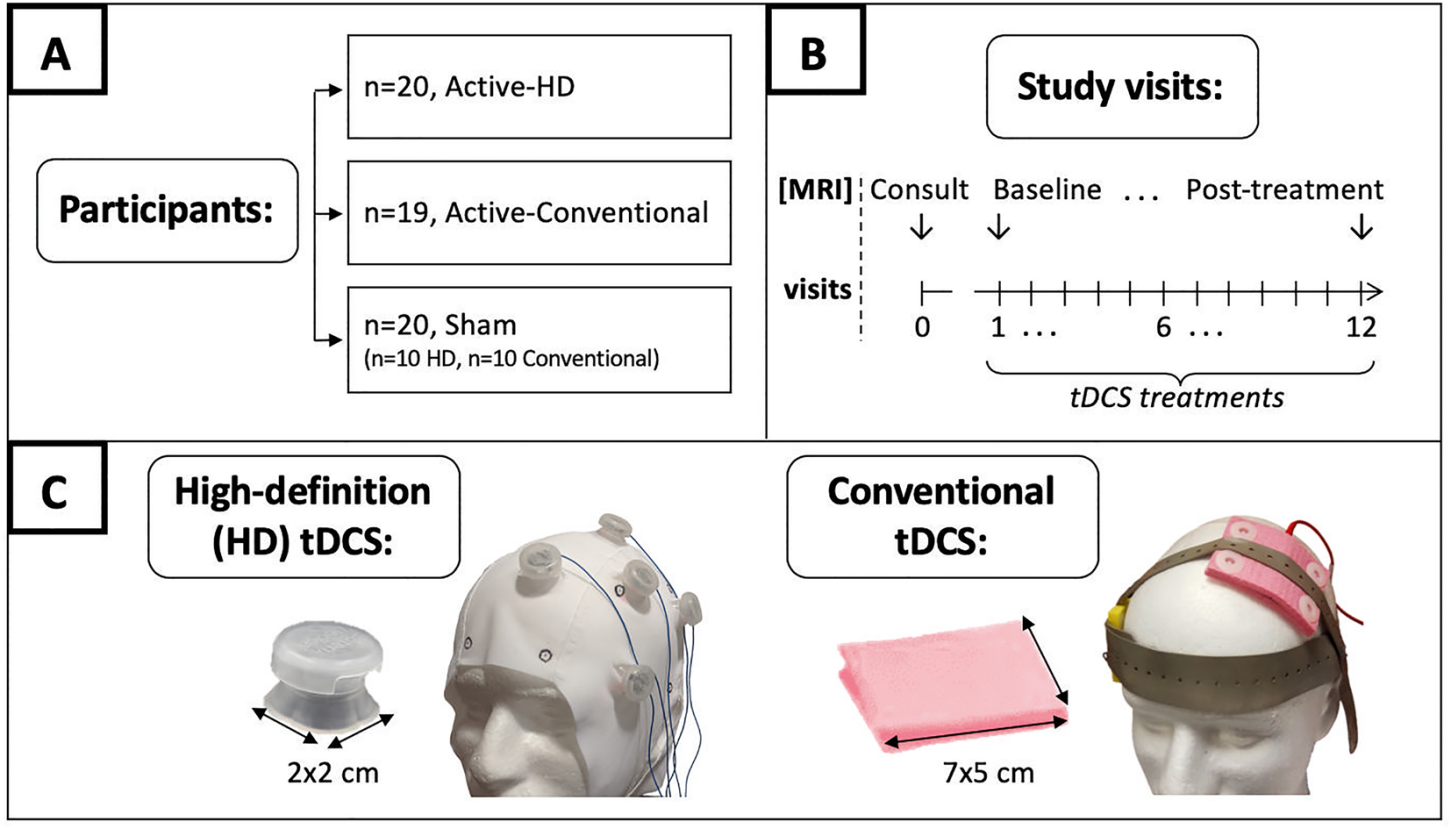
Study diagram. (**A**) In this randomized, double-blind, sham-controlled study, 59 depressed participants were enrolled and randomized to receive active High Definition (HD) tDCS treatment (n = 20), active conventional tDCS treatment (n = 19), or sham tDCS treatment (n = 10 sham conventional and n = 10 sham HD tDCS) (parallel study design). (**B**) Participation involved a total of 13 visits, with structural MRI data being acquired at the consult, baseline, and post-treatment visits, and tDCS treatments being administered between the baseline and post-treatment visits. (**C**) shows the HD and conventional tDCS setups used to administer tDCS treatments. The HD montage consisted of 2 × 2 cm electrodes in a 4 × 1 ring arrangement, with the central stimulating anode electrode over the left dorsolateral prefrontal cortex (left DLPFC) stimulation target and the return cathode electrodes placed 5 cm away and equidistant from the neighboring two cathode electrodes. The conventional tDCS montage utilized larger 7 × 5 cm electrodes, with the stimulating anodal electrode placed over the left DLPFC stimulation target and the return cathode electrode positioned over the F8 location in the 10–20 EEG coordinate system. Note that both montages were individualized to target the left DLPFC in each participant using the consult visit structural MRI data and neuronavigation (see “Methods”).

Structural MRI data was acquired at baseline and after 12 sessions of tDCS treatment (referred to hereafter as post-treatment) from patients with major depression enrolled in a recently completed randomized, double-blind and sham-controlled clinical trial (Fig. 1A,B). Treatments in the trial were designed to target the left dorsolateral prefrontal cortex (left DLPFC) brain region in depressed participants as informed by previous depression treatment studies^19–22^. Participants were randomized to receive active or sham tDCS treatment using one of two tDCS configurations: (a) a large electrode conventional tDCS montage, or (b) a spatially specific high-definition (HD) tDCS montage (Fig. 1C). The main trial (NCT03556124^23^) succeeded in its objective of demonstrating the targeting and functional modulation of depression-relevant brain regions by tDCS using a priori defined target engagement criteria based on magnetic field and arterial spin labeling (ASL) MRI^12^. Here, we investigate whether active tDCS treatment can induce significant changes in gray matter tissue relative to sham tDCS. Follow-up analyses investigating longitudinal gray matter changes within each group considered separately were also performed.

## Results

No significant differences in clinical or demographic characteristics were observed between the active-HD, active-conventional and sham treatment groups (Supplementary material S1). No significant differences in the mean HAMD scores between treatment groups were observed (mean HAMD scores: active-HD: 17.3, active-conventional: 14.7, sham: 15.0). No significant differences in guesses of active/sham stimulation were observed between the treatment groups for participants or assessors (Participants: $\chi$^2^ = 1.54, *p* = 0.46; Assessors: $\chi$^2^ = 0.045, *p* = 0.97). Additionally, no significant differences in stimulation-related discomfort were observed between treatment groups (*p* = 0.20, Supplementary material S2).

**Figure 2:**
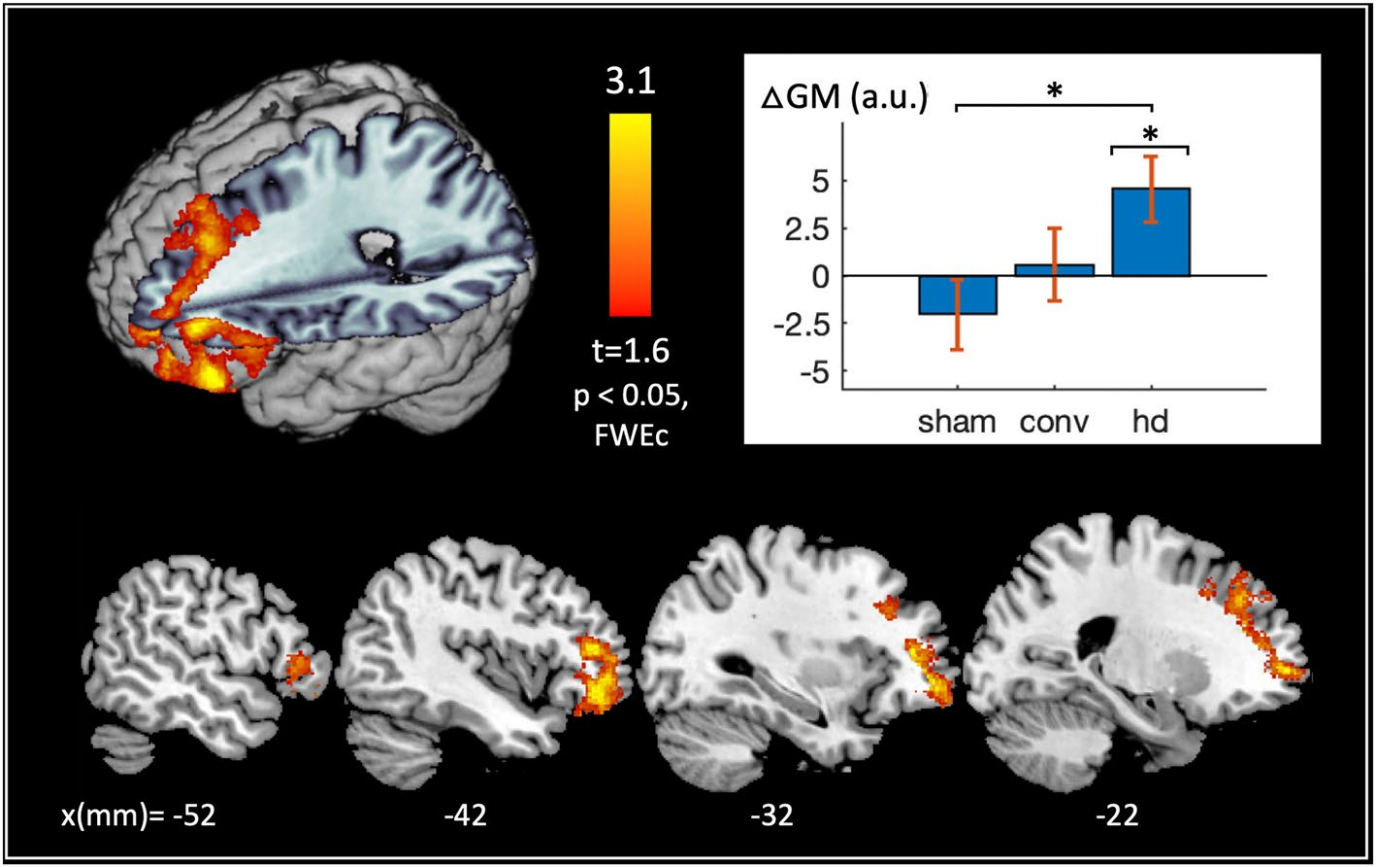
Gray matter changes induced by active tDCS treatment. Gray matter changes (post-treatment–baseline) for the active-HD, active-conventional and sham treatment groups were modeled voxelwise using a 1-way ANOVA with total intracranial volume, age and gender as covariates in SPM12^47^. Planned contrasts were used to investigate gray matter changes induced by active tDCS treatments relative to placebo (i.e., relative to sham tDCS). No significant (family-wise error corrected (FWE) *p* < 0.05 or false discovery rate corrected (FDR) *p* < 0.05, cluster-level) changes in gray matter were observed with active-conventional tDCS relative to sham. In contrast, significant (FWE*p* < 0.05, cluster-level) changes in gray matter were observed with active HD tDCS relative to sham at the left dorsolateral prefrontal cortex (DLPFC) brain region, depicted in the 3D rendering and slice view (with slice locations along the x-axis of the Montreal Neurological Institute (MNI) coordinate system specified in millimeters (mm) at the bottom of the figure). Note that the left DLPFC was also the tDCS stimulation target in this study. Post hoc *t* tests on the cluster-averaged data confirm significant gray matter increases in the active-HD group, and no significant changes in the active-conventional and sham treatment groups post-treatment, as shown in the bar plot (active-HD vs. 0: *p* = 0.010*, d = 0.59; active-conventional vs. 0: *p* = 0.76, d = 0.071; sham vs. 0: *p* = 0.28, d = − 0.24). Abbreviations used in the plot: △GM = cluster-averaged gray matter changes, a.u. = arbitrary units, ‘sham’, ‘conv’, and ‘hd’ = sham, active-conventional and active-HD treatment groups respectively, and * indicates *p* < 0.05.

Significant (*p* < 0.05 FWE, cluster-level) changes in gray matter were observed with active HD tDCS relative to sham at the left DLPFC stimulation target (Fig. 2). Here, post hoc *t* tests on cluster-averaged data revealed significant gray matter increases in the active-HD group post treatment and no significant changes in the sham or active-conventional groups (active-HD vs. 0: *p* = 0.010, Cohen’s *d* = 0.59; active-conventional vs. 0: *p* = 0.76, *d* = 0.071; sham vs. 0: *p* = 0.28, *d* = − 0.24). An additional post hoc 1-way ANOVA was used to test for differences in pre-treatment gray matter volume within the significant left-DLPFC cluster; here, no significant differences were observed between the three treatment groups (*p* > 0.8).

No significant (FWE*p* < 0.05, cluster-level) differences between the active-conventional and sham treatment groups were observed within the cerebrum; similarly, no significant differences were noted using a more lenient false discovery rate criterion (FDR*p* < 0.05, cluster-level).

**Figure 3:**
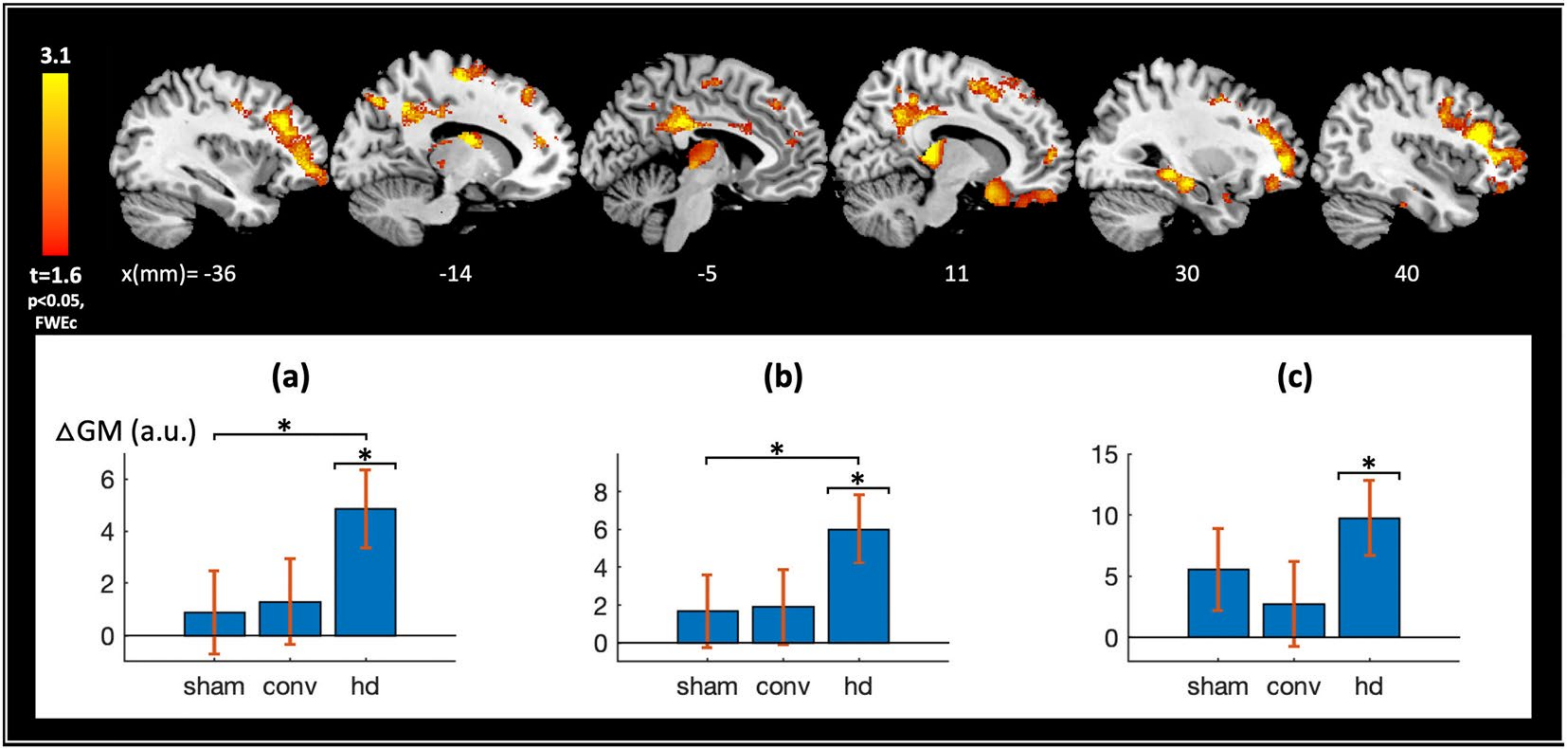
Gray matter changes within individual treatment groups. A follow-up analysis was performed to investigate longitudinal gray matter changes within each individual treatment group. This provides a methodological check, since no significant changes in gray matter are expected to result from sham tDCS treatment. Additionally, significant regions identified in the active tDCS groups, though not dissociable from placebo effects in this analysis, can be used for defining a priori brain regions for future datasets investigating tDCS induced structural changes. No significant (family-wise error corrected (FWE) *p* < 0.05 or false discovery rate corrected (FDR) *p* < 0.05, cluster-level) changes in gray matter were detected in the sham treatment group (as expected), nor in the active-conventional group. In contrast, significant changes were detected in the active-HD group in three clusters comprising the (**a**) left dorsolateral prefrontal cortex (DLPFC) and bilateral posterior cingulate cortex (FWE*p* < 0.05, cluster-level), (**b**) the right DLPFC and subgenual cingulate cortex (FWE*p* < 0.05, cluster-level), and (**c**) the right hippocampus, thalamus and left caudate brain regions (FDR*p* < 0.05, cluster-level). These results are depicted in the slice view, with slice locations along the x-axis of the Montreal Neurological Institute (MNI) coordinate system specified in millimeters (mm) at the bottom of the figure. (**a**–**c**) show post hoc *t* tests performed on the structural data from (**a**–**c**) clusters (defined above), and confirm significant gray matter increases with treatment in the active-HD group in each cluster, and no significant changes in the active-conventional and sham treatment groups (active-HD vs. 0: “*p*_(a)_” = 0.0020*, *d*_(*a*)_ = 0.73; “*p*_(b)_” = 0.0016*, *d*_(*b*)_ = 0.74; “*p*_(c)_” = 0.0028*, *d*_(*c*)_ = 0.70; active-conventional vs. 0: *p*_(a)_ = 0.44, *d*_(*a*)_ = 0.18; *p*_(b)_ = 0.35, *d*_(*b*)_ = 0.22; *p*_(c)_ = 0.43, *d*_(*c*)_ = 0.19; sham vs. 0: *p*_(a)_ = 0.58, *d*_(*a*)_ = 0.12; *p*_(b)_ = 0.39, d_(b)_ = 0.19; *p*_(c)_ = 0.10, d_(c)_ = 0.37). Note that since the (**a**–**c**) clusters were defined by thresholding an active-HD vs. 0 statistical map, the associated active-HD vs. 0 cluster “*p* values”, provided solely for contextual comparison to those found in the other groups, are listed in quotation marks. Abbreviations used in the plot: △GM = cluster-averaged gray matter changes, a.u. = arbitrary units, ‘sham’, ‘conv’, and ‘hd’ = sham, active-conventional and active-HD treatment groups respectively, and * indicates *p* < 0.05.

Figure 3 shows the results of the follow-up analysis investigating significant post-treatment gray matter changes within each individual treatment group. No significant changes were observed within the sham or active-conventional groups. In contrast, significant (FWE*p* < 0.05 cluster-level) gray matter changes were observed in the active-HD group in two distinct clusters including (a) the left DLPFC and bilateral posterior cingulate cortex and (b) the right DLPFC and subgenual anterior cingulate cortex brain regions. An additional cluster (c) comprising the right hippocampus, thalamus and left caudate regions survived the relatively more lenient FDR*p* < 0.05 (cluster-level) significance threshold. Figure 3 bar plots summarize the observed neurostructural changes using cluster-averaged data across each treatment group. Significant gray matter increases were observed in the active-HD group, and no significant changes were observed in the active-conventional and sham treatment groups for each of the (a)–(c) clusters (active-HD vs. 0: “*p*_(a)_” = 0.0020, *d*_(*a*)_ = 0.73; “*p*_(b)_” = 0.0016, *d*_(*b*)_ = 0.74; “*p*_(c)_” = 0.0028, *d*_(*c*)_ = 0.70; active-conventional vs. 0: *p*_(a)_ = 0.44, *d*_(*a*)_ = 0.18; *p*_(b)_ = 0.35, *d*_(*b*)_ = 0.22; *p*_(c)_ = 0.43, *d*_(*c*)_ = 0.19; sham vs. 0: *p*_(a)_ = 0.58, *d*_(*a*)_ = 0.12; *p*_(b)_ = 0.39, *d*_(*b*)_ = 0.19; *p*_(c)_ = 0.10, *d*_(*c*)_ = 0.37). Since clusters (a), (b) and (c) were defined by thresholding an active-HD vs. 0 statistical map, the associated active-HD vs. 0 cluster “*p* values”, provided solely for contextual comparison to those found in the other groups, are listed in quotation marks.

## Discussion

In this study, we investigated whether serial tDCS treatment administered using conventional or high-definition (HD) setups can induce structural plasticity in depressed participants. Structural MRI data was taken from a recently completed randomized, double-blind and sham-controlled clinical trial in depression that sought to confirm tDCS targeting and functional modulation of depression-relevant brain regions using novel imaging methods that did not include changes in brain structure as a primary hypothesis or criterion for target engagement (NCT03556124^23^, R61 phase). Results from this trial demonstrated accurate delivery of tDCS at the left DLPFC stimulation target (through MRI measurements of the tDCS current-induced magnetic field) and post-treatment functional modulation of the left DLPFC stimulation target and the anterior cingulate cortex through MRI measurements of cerebral blood flow changes^12^. In this same cohort where targeting and functional modulation of the left DLPFC by conventional and HD tDCS had already been empirically demonstrated, we observed in the current study significant treatment-induced gray matter increases at the left DLPFC stimulation target after a 12-day course of HD tDCS therapy.

Approximately half of the patients in each treatment group were receiving stable standard antidepressant medications. The number of such patients did not differ significantly between the groups (Supplementary material S1) and the medication status of these patients was unchanged 6 weeks prior to and during their participation in this trial. Additionally, a post hoc region-of-interest analysis that excluded structural data from participants taking antidepressants showed that the patterns of structural changes across treatment groups remained similar for each significant region identified by the study analyses (see Supplementary material S3). Note that tDCS treatment was administered by study staff without any concurrent adjunctive experimental intervention. Blinding integrity was verified, and no significant differences in stimulation-related discomfort were observed between the treatment groups. Therefore, our results demonstrating significant HD tDCS induced gray matter changes cannot be attributed to these potential confounds, nor to the additive effects of an adjunct treatment, and are unlikely to be due to tDCS augmentation of ongoing antidepressant therapy. Overall, our findings indicate that 2 mA × 20 min state-of-the-art HD tDCS treatment protocols can induce long-term neurostructural changes in human subjects, despite potentially only a fraction of the applied tDCS current reaching the cortex^11^. These findings are especially salient for depression, where mixed results from recent clinical trials^7,8^ have emphasized the need to demonstrate targeting and modulation of depression-relevant neural circuitry by tDCS treatment protocols.

Treatment-induced structural changes have been reported with other electromagnetic neuromodulation modalities. For example, repetitive transcranial magnetic stimulation (rTMS) studies targeting the same left DLPFC stimulation target in depressed participants have reported treatment-induced structural changes in cortical and subcortical brain regions including the cingulate gyrus and hippocampus^24,25^. Robust volumetric increases in the hippocampus and amygdala have been reported by multiple studies employing electroconvulsive therapy (ECT) in depression^26–28^, with less pronounced gray matter volume increases also observed in a distributed fashion across many other brain regions^29^.

The cellular basis of these electromagnetic treatment-induced morphological changes is not well understood. In ECT, the administered large strength, short duration electric currents are designed to induce seizures^30,31^, and are hypothesized to induce structural changes through a combination of treatment-induced neurogenesis in the hippocampus and changes in synaptic density, glial cell numbers and angiogenesis as described in^32^. While neurogenesis is unlikely to be the cause of the tDCS induced cortical changes observed in this study, the low-strength, long duration electric currents of tDCS have been shown recently to induce persistent changes in dendritic spine density in rats^16^, and may induce changes in glial structure^33^. These processes may contribute to the volumetric changes induced by serial tDCS in humans; however, the exact microstructural nature of these changes cannot be determined with the present MRI dataset and requires further investigation.

### Montage-specific differences

In this study, serial HD tDCS treatment induced significant neurostructural changes in depressed participants. No significant changes were observed with treatments administered using the conventional tDCS montage. This pattern of montage-specific changes was also observed in the follow-up analyses investigating post-treatment structural changes within each individual treatment group. Although not compared directly to sham, results suggest that HD-tDCS induces changes in the cortico-thalamic-striatal-limbic circuitry widely implicated in depression treatment studies^34,35^. Note that no significant differences in stimulation-related discomfort were observed between the two active treatment groups (Supplementary material S2). Furthermore, for both groups, the applied tDCS was identical (= 2 mA × 20 min over 12 consecutive working days), and the simulated electric current as well as the tDCS current-induced magnetic field measurements at the left DLPFC stimulation target were comparable (reported in^12^). Taken together, these results indicate that the observed montage-specific differences result from dose characteristics outside the left DLPFC stimulation target. In particular, the bihemispheric electrode arrangement of the conventional montage (consistent with typical montages used in depression studies^2^) likely results in the targeting of additional brain regions beyond the left DLPFC (also predicted by simulations, see^36^). Overall, our findings of montage-specific gray matter changes suggest that *spatially* *specific* modulation of the left DLPFC is more effective at inducing structural plasticity in depression.

In contrast to the montage-specific structural changes at the left DLPFC, comparable functional changes were induced by conventional and HD tDCS in the same region in the same cohort (demonstrated using ASL-MRI cerebral blood flow data, and reported in^12^). The different patterns of structural and functional changes indicate that such effects may not be perfectly aligned in terms of their mechanisms or timing. Consequently, we recommend the acquisition of structural data in future neuroimaging studies of tDCS to facilitate the mapping of this understudied phenomenon. An understanding of both structural and functional changes is critical to advance our understanding of tDCS mechanisms and optimize treatment protocols for clinical applications.

### Clinical relevance

A significant negative correlation was observed between %-change (post–pre treatment) HAMD and post-treatment changes in gray matter averaged over the significant cluster identified in the primary analysis (r = − 0.28, *p* = 0.048, Supplementary material S4), indicating that tDCS treatment-induced neurostructural changes are associated with improvements in mood. A trend was also observed between post-treatment gray matter changes and %-change in the Snaith Hamilton Pleasure scale (SHAPS^37^; r = − 0.29, *p* = 0.052). The SHAPS metric measures anhedonia (a core symptom of depression), and has previously been shown to improve with tDCS treatment in the same sample^12^. No statistically significant improvements were observed in the HAMD and HAMD-6 scores between the active treatment and sham groups (Supplementary material S5). However, it should be noted that the current arm of our clinical trial (NCT03556124^23^, R61 phase) was not designed or powered to investigate efficacy, but rather to evaluate tDCS targeting and functional modulation at a priori selected depression-relevant brain regions. Additionally, mood scores were only measured at the baseline and post-treatment timepoints, which are approximately 2 weeks apart. Recent studies investigating tDCS therapy in depression have indicated that the clinical effects of tDCS may be delayed^38^ and have reported significant mood improvement (relative to placebo) only after week 6^7,39^.

Besides mood changes, depression is also associated with widespread reductions in gray matter in regions including the DLPFC^14,15,40^, cingulate cortex (including the anterior^14,15,41^, posterior^40,41^ and subgenual^42^ cingulate cortices), hippocampus, thalamus, and amygdala^14,15,26,43^. Our primary analysis indicated that HD tDCS treatment induces significant gray matter increases at the left DLPFC brain region. Follow-up analyses in individual groups showed gray matter increases in the left and right DLPFC, posterior and subgenual cingulate cortices, hippocampus, thalamus and caudate with HD tDCS treatment (although the effects could not be dissociated from placebo in regions other than the left DLPFC). Overall, the depression research literature indicating gray matter *reductions* in these regions and our findings suggesting HD tDCS treatment induced gray matter *increases* in the same regions indicates that serial HD tDCS treatment may partly normalize the structural pathology reported in major depression.

### Limitations and future directions

This study has several limitations. First, mood scores were not measured beyond the post-treatment timepoint, such that it was not possible to evaluate antidepressant effects (which recent studies indicate may improve for up to 6 weeks after tDCS) and their relationship with the observed treatment-induced neurostructural changes. A second limitation of our study is the relatively small sample size of each individual treatment group (n ≤ 20), which limits the sensitivity of the analyses performed. Although widespread gray matter increases were observed within the active HD treatment group in the follow-up analyses (due to its more powerful within-subjects design), these could not be dissociated from placebo effects except within the left DLPFC brain region. Even so, the latter results provide a rational means for future studies to restrict an otherwise whole-brain voxelwise analysis to specific regions, to conserve statistical power and improve the sensitivity of analyses. For example, we plan to use the brain regions identified in this study to investigate structural changes in our follow up study arm that involves data acquisition in N = 100 depressed participants receiving active or sham HD tDCS (NCT03556124^23^, R33 phase). This study arm also includes the acquisition of behavioral data for up to 4 weeks beyond the post-treatment timepoint, which is expected to facilitate the study of neurostructural effects relative to behavioral changes. Finally, whole-brain voxelwise analysis used in this study allowed us to investigate tDCS-related changes in macrostructure. A lack of prior studies examining structural changes associated with tDCS precluded the a priori definition of specific regions for a more focal region-of-interest analysis.

## Methods

### Study participants

A total of 305 depressed participants were assessed for eligibility, of whom 66 were enrolled and 59 (32 F) completed treatment. All participants provided informed consent following approval of study procedures by the University of California Los Angeles (UCLA) Institutional Review Board (IRB). All methods and study procedures were performed in accordance with the relevant guidelines and regulations of the UCLA IRB.

Enrolled participants were required to meet the criteria for a current major depressive episode which was confirmed using the Structured Clinical Interview for DSM-5^44^. Additionally, included participants were required to (a) have a Hamilton Depression Rating Scale^45^ (HDRS) 17-item score ≥ 14 and < 24 assessed at the consult visit (Fig. 1), (b) be between 18 and 55 years old, and (c) be treatment naïve, or on a stable standard antidepressant regimen (including selective serotonin reuptake inhibitors (SSRIs), serotonin-noradrenaline reuptake inhibitors (SNRIs), monoamine oxidase inhibitors (MAOIs) or tricyclics (TCAs)) with no change in treatment 6-weeks prior to and during the tDCS intervention. Participants with severe or treatment-resistant depression (HAMD scores ≥ 24 and a history of a major depressive episode lasting > 2-years or failure to 2 or more antidepressant trials in the current index episode) were excluded. The full list of exclusion criteria is described in Supplementary material S6.

### Experimental design

A randomized, double-blinded, parallel study design was employed. As shown in Fig. 1, enrolled study participants were randomized to receive active or sham tDCS treatment using one of two tDCS configurations: (a) a large electrode conventional tDCS montage, or (b) a spatially specific high-definition (HD) tDCS montage.

### tDCS

The left dorsolateral prefrontal cortex (left DLPFC) brain region, a key node in the top-down mood-regulating circuitry frequently implicated in depression^21,22^ was selected to be the stimulation target. Specifically, tDCS configurations were designed to target the [− 46, 44, 38] mm standardized Montreal Neurological Institute (MNI) coordinate in the left DLPFC based on prior studies^19,20^. This location was individualized in each participant as follows: First, structural MRI data was acquired at the consult visit (visit #0, see Fig. 1) using the Lifespan Human Connectome Project T1-weighted MPRAGE sequence (^46^, sequence parameters: TE1/TE2/TE3/TE4 = 1.81/3.6/5.39/7.18 ms, TI = 1 s, TR = 2.5 s, 8^0^ FA, 0.8 × 0.8 × 0.8 mm^3^ voxel, 320 × 320 matrix, 208 slices, 740 Hz/Px bandwidth for all TE’s, 6/8 partial Fourier, R = 2 GRAPPA acceleration) on a Siemens 3 T PRISMA scanner using a 64-channel coil. Next, the stimulation target MNI coordinates were transformed into the individual subject’s coordinate space using inverse normalization (implemented in SPM12^47^). Finally, frameless stereotaxy neuronavigation and associated software (Brainsight^48^) was used to locate the individualized stimulation target in each subject. The neuronavigation system uses fiducial markers and a probe to establish a mapping between landmarks on the participant’s head and their location in the participant’s consult visit digital MRI image. Once established, this mapping was used to accurately locate the individualized stimulation target coordinates that were calculated in the previous step, on the participant’s scalp. The coordinates were marked on a carefully placed and tight-fitting cap, and the marked cap was used for positioning the tDCS electrodes on all subsequent study visits. This methodology of using state-of-the-art neuronavigation equipment for identifying personalized stimulation targets and careful cap placement procedures for ensuring accurate and reliable electrode positioning for serial tDCS treatments has been previously validated^49^.

For HD tDCS, a 4 × 1 setup was used with the central stimulating anode electrode placed over the stimulation target and the cathode electrodes placed 5 cm away and equidistant from the neighboring cathodes. For conventional tDCS, 5 × 7 cm-sized electrodes were employed, with the anode placed over the stimulation target and the cathode placed over [56, 30, − 1] mm MNI coordinates (~ approximately F8 in the 10–20 coordinate system). Each enrolled participant received 12 sessions of active or sham tDCS (depending on the randomization) over 12 consecutive working days. Active tDCS involved 2 mA × 20 min of tDCS, including 30 s ramp-times. Sham tDCS involved a 30 s ramp up to 2 mA followed by a 30 s ramp down to zero at the beginning of the 20 min session. Apart from this ramp up/down period, ammeter readings showed that the stimulator emitted a steady current of 65 µA during sham tDCS. This current intensity is an order of magnitude smaller than the intensity employed in active tDCS and is similar to sham-tDCS utilized by^8^.

To maintain blinding, each participant was assigned a unique code upon enrollment, and this code was used by study staff to operate the double-blind stimulator device (Soterix Model #5100D). Both participants and assessors were asked to guess the stimulation condition (active/sham) at the study endpoint to verify integrity of blinding. Data on stimulation-related discomfort was also acquired using the Generic Assessment of Side Effects (GASE) scale^50^.

### Data processing

Longitudinal structural MRI data was acquired at baseline and after 12 sessions of tDCS treatment (i.e., post-treatment). This data was acquired using the same MRI sequence as used during the consult visit MRI scan described above. The acquired data was skull-stripped and segmented using voxel-based morphometry (VBM^51^, implemented in CAT12^52^) to estimate voxelwise gray matter volume maps. Next, difference maps (post-treatment–baseline) were calculated, smoothed (8 mm gaussian kernel), and masked using a SPM12^47^ gray matter tissue probability mask thresholded at 0.2 and excluding the cerebellum.

The baseline and post-treatment MRI sessions also involved the acquisition of concurrent tDCS/MRI data (the latter was analyzed and reported in^12^). All structural data was acquired before the concurrent tDCS/MRI scans, precluding any potential acute effects of tDCS stimulation in the observed results. The acquisition of concurrent tDCS/MRI data also made it necessary for all participants to wear a cap during the MRI sessions, to secure the tDCS electrodes to the scalp. The presence of caps and electrodes is known to induce systematic biases in gray matter volume estimation^53^; however, these biases are controlled for in the present analyses by the post-treatment–baseline differencing step. We verified that there were no longitudinal differences in the position of the tDCS stimulating electrode between treatment groups to ensure this subtraction did not influence results (Supplementary material S7).

### Statistical analysis

Maps of longitudinal differences in gray matter volume (calculated using the method described in the data processing sub-section above) were modeled voxelwise using a 1-way ANOVA with the total intracranial volume (TIV), age and gender as covariates in SPM12 as recommended by CAT12^52^. We hypothesized that active tDCS treatment would induce significant changes in gray matter macrostructure and investigated this question using two planned t-contrasts (active-HD vs. sham, and active-conventional vs. sham). Results were thresholded at *p* < 0.05, and corrected for multiple comparisons at the cluster level using family-wise error correction (FWE) or the more lenient false discovery rate (FDR) correction at *p* < 0.05, implemented in SPM12^47^.

Follow-up analyses investigating longitudinal gray matter changes within each group considered separately were also performed. Though not able to disentangle treatment effects from potential placebo effects, the more powerful within-subjects design of these secondary analyses permits identification of brain regions that could be used for generating a priori hypotheses in future studies. Currently lacking such a priori anatomical hypotheses, analyses in the present study investigated tDCS induced structural changes at each voxel within the cerebrum since the effects of tDCS can potentially propagate neuronally through brain networks^54^.

Differences in clinical and demographic characteristics between the treatment groups were investigated using a 1-way ANOVA (for continuous random variables) and a $\chi$^2^ test (for categorical variables). To assess blinding integrity, a $\chi$^2^ test was used to investigate differences in guesses of stimulation condition (active/sham) between treatment groups in both participants and assessors. Finally, stimulation related items in the GASE scale were averaged over the 12 tDCS treatment sessions and compared using a 1-way ANOVA to investigate differences in stimulation-related discomfort between treatment groups.

## Supplementary Information

Supplementary Information.

## Data Availability

The data is available on the NIMH data archive (https://nda.nih.gov/edit_collection.html?id=2737).
