# Supplementary material for: Transcranial direct current stimulation (tDCS) in depression induces structural plasticity
Source: Sci Rep. 2023 Feb 17;13:2841. doi: 10.1038/s41598-023-29792-6 (PMC9938111; doi:10.1038/s41598-023-29792-6)
Supplement: Supplementary file 1 — Supplementary Information. [file 41598_2023_29792_MOESM1_ESM.pdf]

Supplementary Information for

Transcranial direct current stimulation (tDCS) in depression induces structural plasticity.

Mayank .A. Jog[1] , Cole Anderson[2], Antoni Kubicki[1], Michael Boucher[3], Amber Leaver[4], Gerhard Hellemann[5], Marco Iacoboni[6], Roger Woods[1,6], Katherine Narr[1,6,*]

[1] Department of Neurology, University of California Los Angeles (UCLA), Los Angeles, CA, 90095
[2] Diagnostic Imaging Sciences Center, University of Washington, Seattle, WA, 98195
[3] Semel Institute for Neuroscience and Human Behavior, UCLA, Los Angeles, CA, 90095
[4] Department of Radiology, Northwestern University, Evanston, IL, 60208
[5] Department of Biostatistics, University of Alabama at Birmingham, Birmingham, AL, 35294
[6] Department of Psychiatry and Biobehavioral Sciences, UCLA, Los Angeles, CA, 90095

*Corresponding author: Katherine Narr.
Email: knarr@mednet.ucla.edu

This PDF file includes:

S1: Clinical and demographic characteristics of participants
S2: Stimulation-related discomfort
S3: Post hoc analysis excluding structural data of participants taking antidepressants
S4: Correlation between longitudinal changes in gray matter and behavioral changes
S5: Treatment-induced changes in mood
S6: Full list of exclusion criteria
S7: Relative electrode positions between the baseline and post-treatment structural data
SI References

S1: Clinical and demographic characteristics of participants

Table S1 shows the clinical and demographic characteristics of the depressed participants overall (column 1), and within each tDCS treatment group (columns 2-4). Between-group differences in subject characteristics were tested using a 1-way ANOVA (for continuous random variables) and χ^2-tests (for categorical random variables) and are shown in column 5. No significant differences between groups were observed for any clinical or demographic characteristic.

	Total (N=59)	Groups			Between-groups differences (p)
		Sham (n=20)	Active-Conv (n=19)	Active-HD (n=20)	
**Characteristics**					
Females (%)	54.2	60.0	42.1	60.0	0.44
⸭Age, yrs.	31.1 (8.34)	30.9 (7.46)	30.7 (9.98)	31.6 (7.88)	0.94
⸭ Years at school	17.7 (3.16)	18.2 (2.34)	16.6 (4.61)	18.1 (1.81)	0.21
⸭BMI	24.6 (4.94)	23.9 (4.28)	25.8 (6.45)	24.1 (3.74)	0.44
**Clinical characteristics**					
⸭ Onset age, yrs.	15.4 (5.81)	17.3 (6.13)	13.6 (4)	15.1 (6.56)	0.17
Unipolar depression	58	19	19	20	0.37
Bipolar (Type II)	1	1	0	0	0.37
**Antidepressant drugs**					
No antidepressants	33	14	8	11	0.21
SSRI monotherapy	19	3	9	7	0.09
Dual antidepressant therapy	5	1	1	3	0.44
Other (SNRI or atypical monotherapy)	8	2	3	3	0.85

Table S1: Clinical and demographic characteristics. Table S1 shows the clinical and demographic characteristics overall (column 1) and within sham, active conventional and active HD treatment groups (columns 2-4). Quantities marked with a ⸭ are reported as Mean (SD), and total counts are reported for the remainder. No significant differences in any of the characteristics were observed between groups (tested with a 1-way ANOVA for continuous random variables and χ^2-tests for categorical random variables; note the p-values reported in column 5). SSRI = Selective Serotonin Reuptake Inhibitor and SNRI = Serotonin and norepinephrine reuptake inhibitor. Some enrolled participants were on two (stable) antidepressant medications and were categorized into a separate 'dual antidepressant therapy' group for analyses. None of the enrolled participants were on more than two medications. The atypical category included participants on buproprion and trazodone. Participants using anticonvulsants, lithium, psychostimulants and dexamphetamine were excluded, as were participants with bipolar I disorder (due to possible risk of mania and because lithium and anticonvulsants were also exclusionary criteria). The full inclusion/exclusion criteria are described in Methods and Supplementary material S4.

S2: Stimulation-related discomfort

The Generic Assessment of Side Effects (GASE) data [1] was acquired after each study visit for every participant. Stimulation-related discomfort was assessed by first collecting all the stimulation-relevant items from the GASE data (including headache, dizziness, palpitations, breathing difficulty, nausea, rash, fever, and fatigue) followed by an averaging of scores across items and visits for each participant to calculate a modified GASE score. These modified GASE scores, representative of stimulation-related discomfort, were subsequently tested for differences between treatment groups using a 1-way ANOVA. Shown in Figure S1, no significant differences were observed (p = 0.20).

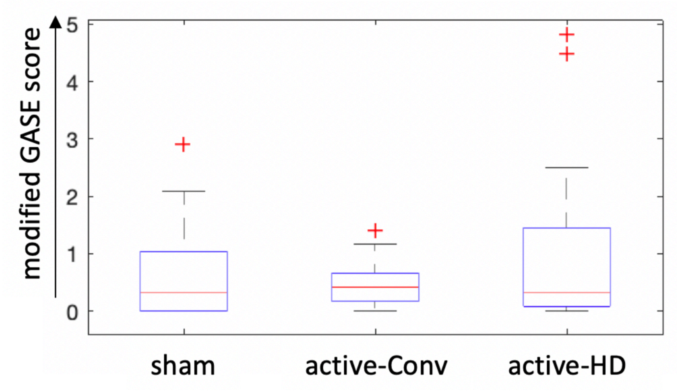

Figure S1: Stimulation-related discomfort. Stimulation-related discomfort was assessed using a modified GASE score (see text for details) for each participant in the sham, active-conventional and active-HD treatment groups. Scores are shown in the figure using a box-plot, note that the red lines and edges of the box indicate median discomfort and 25/75 percentiles respectively. In the active-HD group, two subjects were observed to feel more discomfort compared to the others. Even so, no systematic difference between groups was observed with a 1-way ANOVA using all of the data (p = 0.20). When the outliers were excluded, systematic differences between groups remained non-significant (p=0.54).

S3: Post hoc analysis excluding structural data of participants taking antidepressants
Average gray matter changes within the significant region identified by the primary analysis (reported in Figure 2 of the main manuscript) were investigated using structural data from participants that did not take any antidepressants over the duration of the trial. As shown in Figure S2, identical patterns of significance and similar effect-sizes were observed when analyses utilized data exclusively from antidepressant-free participants instead of the original sample.

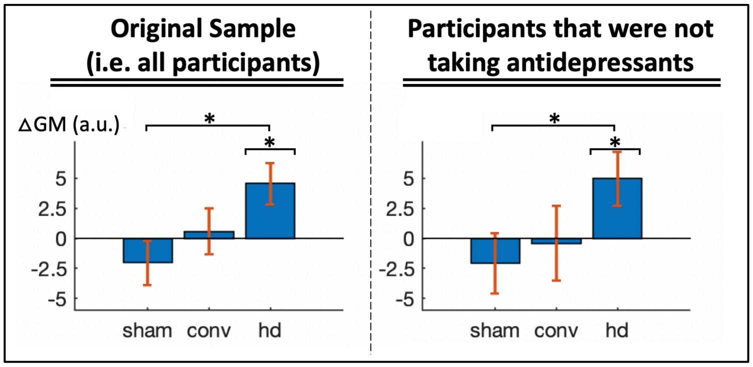

Figure S2. Effects of excluding participants on antidepressants: Barplot on the left shows the average gray matter change within each treatment group in the left dorsolateral prefrontal cortex brain region that was identified by the primary analysis in the main manuscript (active-HD vs. 0: p=0.010*, *d*=0.59; active-conventional vs. 0: p=0.76, *d*=0.071; sham vs. 0: p=0.28, *d*=-0.24; note that this plot is identical to the barplot of Figure 2, and is shown here for ease of comparison). Barplot on the right shows the same measured quantities, albeit calculated using structural data from participants that did not take any antidepressants during the course of the trial (active-HD vs. 0: p=0.035*, *d*=0.50; active-conventional vs. 0: p=0.90, *d*=-0.03; sham vs. 0: p=0.42, *d*=-0.18). As can be seen, the patterns of significance are identical and the effect-sizes are similar (difference in

cohen's *d* < 0.11). Abbreviations used in the plot: △GM = average gray matter changes, a.u. = arbitrary units, 'sham', 'conv', and 'hd' = sham, active-conventional and active-HD treatment groups respectively, and * indicates p < 0.05.

The same *post hoc* analysis approach was applied to the significant regions identified by the follow-up analysis (i.e. clusters (a)-(c) defined in Figure 3). Here too, similar patterns of significance and effect-sizes were observed (**Analysis performed using the original sample of participants:** active-HD vs. 0: $p_{(a)}$=0.0020*, $d_{(a)}$=0.73; $p_{(b)}$=0.0016*, $d_{(b)}$=0.74; $p_{(c)}$=0.0028*, $d_{(c)}$=0.70; active-conventional vs. 0: $p_{(a)}$=0.44, $d_{(a)}$=0.18; $p_{(b)}$=0.35, $d_{(b)}$=0.22; $p_{(c)}$=0.43, $d_{(c)}$=0.19; sham vs. 0: $p_{(a)}$=0.58, $d_{(a)}$=0.12; $p_{(b)}$=0.39, $d_{(b)}$=0.19; $p_{(c)}$=0.10, $d_{(c)}$=0.37; **Analysis performed after excluding participants on antidepressants:** active-HD vs. 0: $p_{(a)}$=0.0060*, $d_{(a)}$=0.63; $p_{(b)}$=0.01*, $d_{(b)}$=0.63; $p_{(c)}$=0.002*, $d_{(c)}$=0.79; active-conventional vs. 0: $p_{(a)}$=0.93, $d_{(a)}$=0.02; $p_{(b)}$=0.33, $d_{(b)}$=0.23; $p_{(c)}$=0.32, $d_{(c)}$=0.24; sham vs. 0: $p_{(a)}$=0.78, $d_{(a)}$=0.06; $p_{(b)}$=0.54, $d_{(b)}$=0.14; $p_{(c)}$=0.38, $d_{(c)}$=0.20; max. difference in cohen's *d* on * groups < 0.11).

S4: Correlation between longitudinal changes in gray matter and behavioral changes
The primary analysis in this study revealed significantly different longitudinal changes in gray matter at the left dorsolateral prefrontal cortex brain region between the active-HD and sham tDCS treatment groups (see Figure 2 in the main manuscript). Here we investigated correlations between average gray matter changes in this region and %changes (post-pre treatment) in the following behavioral metrics: the Snaith Hamilton Pleasure scale (SHAPS [2]), and the Hamilton Depression Rating scale (HAMD and HAMD-6) [3]. All correlations controlled for age, gender and total intracranial volume, consistent with the recommendations of the Computational Anatomy Toolbox (CAT12)[4].

As shown in Figure S3, significant correlations were observed between post-treatment gray matter changes and %ch-HAMD (r=-0.28, p=0.048). A trend (r=-0.29, p=0.052) was also observed between gray matter changes and %ch-SHAPS. In both cases, post-treatment increases in gray matter were associated with improvements in behavioral scores.

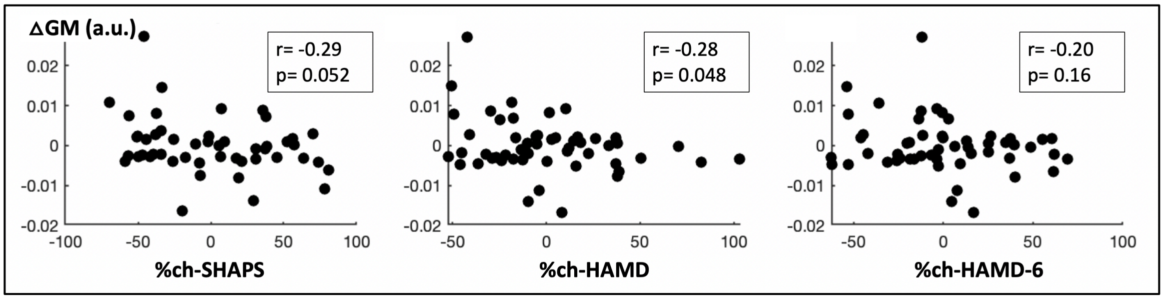

Figure S3. Correlation between post-treatment changes in gray matter and %changes (post-pre treatment) in the Snaith Hamilton Pleasure scale (SHAPS [2]), and the Hamilton Depression Rating scale (HAMD and HAMD-6) [3]: Significant correlations were observed between gray matter changes and %ch-HAMD (r=-0.28, p=0.048), and a trend was observed between gray matter changes and %ch-SHAPS (r=-0.29, p=0.052). No significant correlations were observed between gray matter changes and %ch-HAMD-6. Correlations were performed after pooling gray matter data across treatment groups, and controlled for age, gender and total intracranial volume (consistent with the recommendations of the Computational Anatomy Toolbox (CAT12)[4]). △GM refers to cluster-averaged gray matter changes in arbitrary units (a.u.).

S5: Treatment-induced changes in mood

Figure S4 shows tDCS treatment-induced changes in mood, as assessed using the Hamilton Depression Rating Scale [3]. %-changes in HAMD and HAMD-6 (calculated as post-treatment relative to baseline) were estimated for each treatment-group and compared using a 1-way ANOVA. No significant differences between active HD, active conventional and sham groups were observed for HAMD or HAMD-6. Note that the response rates were measured to be 30%, 26.3% and 40% for the sham, active-conventional and active-HD treatment groups respectively, and the remission rates were measured to be 25%, 10.5% and 25% for the same (responders were defined as % of subjects whose HAMD scores improved by >50%, and remitters were defined as %subjects whose post-treatment HAMD scores were <= 7).

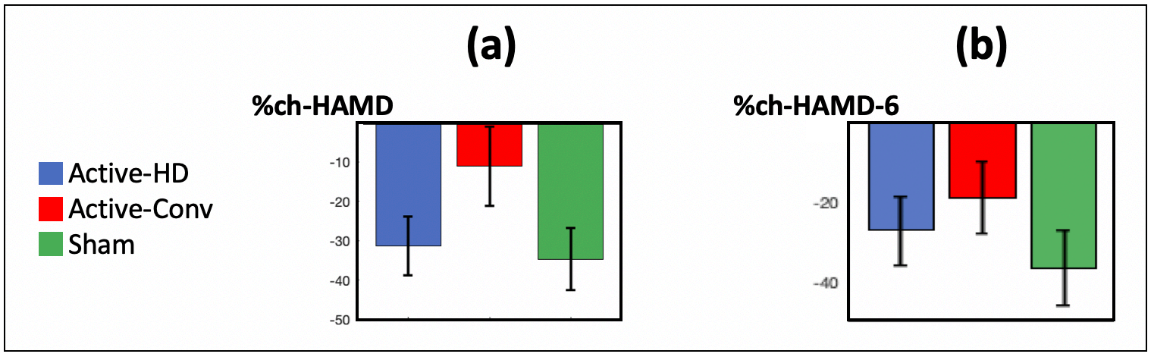

Figure S4: Treatment-induced changes in mood. (a) and (b) show the tDCS induced changes in mood across treatment groups, as assessed using %-change in HAMD and HAMD-6 scores respectively. No significant differences were observed between the active stimulation and sham groups (HAMD: $p_{HD\_vs.\_Sham} = 0.76$, $p_{Conv\_vs.\_Sham} = 0.08$; HAMD-6: $p_{HD\_vs.\_Sham} = 0.47$ $p_{Conv\_vs.\_Sham} = 0.18$); however, this could be because the clinical effects of tDCS are delayed, as indicated by recent studies [5-7] (see discussion/clinical relevance section in the main manuscript).

S6: Full list of exclusion criteria

Exclusion criteria included (a) pregnancy, (b) non-English speaking, (c) substance use disorder within the last 12 months, (d) neurological conditions associated with brain abnormalities (e.g. traumatic brain injury, recent stroke, tumor), (e) contraindications to tDCS (e.g. skin disease or treatment causing irritation), (f) contraindications to MRI (metal implants, claustrophobia or a breathing or movement disorder), (g) currently receiving any form of cognitive behavioral therapy, dialectical behavioral therapy, or acceptance and commitment therapy, (h) change in antidepressant medication within 6 weeks of starting the trial, (i) severe or treatment resistant depression – HAMD scores > 24 and a history of a major depressive episode lasting >2 years or failure to 2 or more antidepressant trials in the current index episode, (j) any neuromodulation therapy (e.g., ECT, rTMS, DBS, VNS or tDCS) within the last 3-months, (k) current or past (within the last 1 month) use of anticonvulsants, lithium, psychostimulant, dexamphetamine, (l) current use of decongestants or other medication including sleeping aids previously shown to interfere with cortical excitability, (m) diagnosis of schizophrenia axis I disorder, or dementia of any type, (n) bipolar I disorder (due to possible risk of mania and because lithium and anticonvulsants are excluded), (o) diagnosis of seizure disorder or history of seizures, (p) depression related to serious medical illness (i.e., mood disorder due to general medical condition), (q) actively suicidal as defined by a score of 4 on item 3 of HAMD, and (r) missing more than 2 study visits.

S7: Relative electrode positions between the baseline and post-treatment structural data
During the acquisition of structural data, participants wore a cap and tDCS electrodes (to facilitate the subsequent acquisition of concurrent tDCS/MRI data, analyzed and reported in [8]). The presence of caps and electrodes has been shown to induce systematic biases in gray matter volume estimation [9]; however, these biases were controlled for in the present analyses by the post-treatment – baseline differencing step. This bias-control would be imperfect if there existed a systematic difference in the position of the tDCS electrode between the baseline and post-treatment structural data, and that this difference was significantly different between active-HD and sham groups (where structural changes were observed in the primary analysis). Consequently, we compared longitudinal differences in electrode positioning between the active-HD and sham groups.

Baseline and post-treatment structural data was realigned to the consult-visit structural data using SPM12 [10] to bring both datasets into the same coordinate space. Next, the central stimulating HD electrode was segmented using ITK-SNAP [11]. The accurately segmented HD electrode mask was used to calculate the center-of-mass coordinate, which was then projected onto the closest scalp voxel. Finally, longitudinal differences in position of this scalp voxel were quantified.

As shown in Figure S5, the estimated longitudinal differences in electrode position were not significantly different between the active and sham HD groups in any direction. The overall electrode displacements were observed to be 6.09±2.87 and 5.67±2.37 mm (mean±SD) in the active and sham groups respectively, with displacements referring to Euclidean distances. Note that these Euclidean displacement values estimated from the structural MRI data closely match the electrode displacements measured using neuronavigation (Brainsight [12]) in the same cohort (6.7±2.7mm, reported in [13], table 1). The neuronavigation sessions were separate from the MRI scanning sessions and had been originally performed at the baseline and mid-trial timepoints to quantify the accuracy and reliability of the electrode placement method employed in this study [13]. The close match between the two measurement approaches provides a further validation of the image processing methodology employed in this supplementary analysis. The conventional tDCS sponge electrodes could not be clearly visualized for segmentation (possibly due to air pockets within the sponge affecting MRI imaging quality). Although noisier, no significant differences in position were observed between active and sham Conventional (data not shown). These observations are consistent with the reproducibility of our electrode positioning technique (documented in [13]).

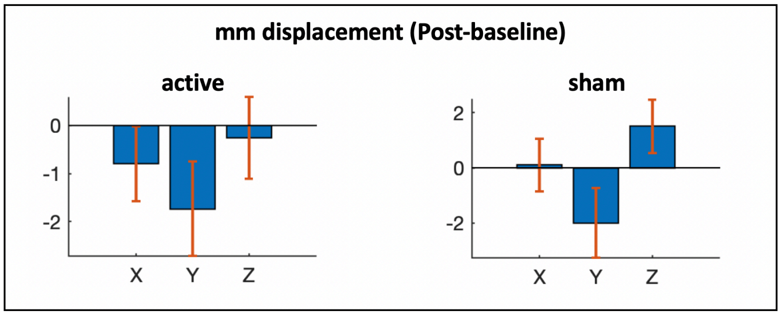

Figure S5. Longitudinal differences in electrode positions. Barplots show the average longitudinal differences in the position of the central stimulating tDCS electrode between the active and sham HD groups along the X, Y and Z spatial directions with error bars representing one standard error. The longitudinal differences were not observed to be significantly different between the two groups for any direction (2-sample t-tests: p_X=0.49, p_Y=0.87, p_Z=0.21). Overall, the electrodes were displaced by 6.09±2.87 and 5.67±2.37 mm (mean±SD) in the active and sham groups respectively, with the displacement values referring to Euclidean distances.

SI References

1 Rief, W. *et al.* Assessing general side effects in clinical trials: reference data from the general population. *Pharmacoepidemiol Drug Saf* **20**, 405-415, doi:10.1002/pds.2067 (2011).

2 Snaith, R. P. *et al.* A scale for the assessment of hedonic tone the Snaith-Hamilton Pleasure Scale. *Br J Psychiatry* **167**, 99-103, doi:10.1192/bjp.167.1.99 (1995).

3 Hamilton, M. A rating scale for depression. *J Neurol Neurosurg Psychiatry* **23**, 56-62, doi:10.1136/jnnp.23.1.56 (1960).

4 Gaser, C. & Dahnke, R. CAT-a computational anatomy toolbox for the analysis of structural MRI data. *Hbm* **2016**, 336-348 (2016).

5 Li, M. S. *et al.* Delayed effect of bifrontal transcranial direct current stimulation in patients with treatment-resistant depression: a pilot study. *BMC Psychiatry* **19**, 180, doi:10.1186/s12888-019-2119-2 (2019).

6 Valiengo, L. C. *et al.* Transcranial direct current stimulation for the treatment of post-stroke depression: results from a randomised, sham-controlled, double-blinded trial. *J Neurol Neurosurg Psychiatry* **88**, 170-175, doi:10.1136/jnnp-2016-314075 (2017).

7 Brunoni, A. R. *et al.* Trial of Electrical Direct-Current Therapy versus Escitalopram for Depression. *N Engl J Med* **376**, 2523-2533, doi:10.1056/NEJMoa1612999 (2017).

8 Jog, M. S. *et al.* In-vivo imaging of targeting and modulation of depression-relevant circuitry by transcranial direct current stimulation: a randomized clinical trial. *Transl Psychiatry* **11**, 138, doi:10.1038/s41398-021-01264-3 (2021).

9 Klein, C., Hanggi, J., Luechinger, R. & Jancke, L. MRI with and without a high-density EEG cap--what makes the difference? *Neuroimage* **106**, 189-197, doi:10.1016/j.neuroimage.2014.11.053 (2015).

10 Penny, W. D. *et al. Statistical parametric mapping: An annotated bibliography.*, <http://www.fil.ion.ucl.ac.uk/spm/bib.htm> (2001).

11 Yushkevich, P. A. *et al.* User-guided 3D active contour segmentation of anatomical structures: significantly improved efficiency and reliability. *Neuroimage* **31**, 1116-1128, doi:10.1016/j.neuroimage.2006.01.015 (2006).

12 Brainbox-Neuro. *Brainsight Neuronavigation*, <https://brainbox-neuro.com/catalogue/neuro-navigation/tms-navigation/brainsight-tms-navigation/> (

13 Jog, M. *et al.* A novel technique for accurate electrode placement over cortical targets for transcranial electrical stimulation (tES) clinical trials. *J Neural Eng* **18**, doi:10.1088/1741-2552/ac297d (2021).
